# Deletion of Type 3 Adenylyl Cyclase Perturbs the Postnatal Maturation of Olfactory Sensory Neurons and Olfactory Cilium Ultrastructure in Mice

**DOI:** 10.3389/fncel.2017.00001

**Published:** 2017-01-19

**Authors:** Zhe Zhang, Dong Yang, Mengdi Zhang, Ning Zhu, Yanfen Zhou, Daniel R. Storm, Zhenshan Wang

**Affiliations:** ^1^College of Life Science, Hebei UniversityBaoding, China; ^2^Medical College, Hebei UniversityBaoding, China; ^3^Department of Cardiology, Baoding First Center HospitalBaoding, China; ^4^Department of Pharmacology, University of Washington, SeattleWA, USA

**Keywords:** olfactory sensory neurons, type 3 adenylyl cyclase, olfactory cilia, main olfactory epithelium, apoptosis

## Abstract

Type 3 adenylyl cyclase (Adcy3) is localized to the cilia of olfactory sensory neurons (OSNs) and is an essential component of the olfactory cyclic adenosine monophosphate (cAMP) signaling pathway. Although the role of this enzyme in odor detection and axonal projection in OSNs was previously characterized, researchers will still have to determine its function in the maturation of postnatal OSNs and olfactory cilium ultrastructure. Previous studies on newborns showed that the anatomic structure of the main olfactory epithelium (MOE) of *Adcy3* knockout mice (*Adcy3*^-/-^) is indistinguishable from that of their wild-type littermates (*Adcy3*^+/+^), whereas the architecture and associated composition of MOE are relatively underdeveloped at this early age. The full effects of sensory deprivation on OSNs may not also be exhibited in such age. In the present study, following a comparison of postnatal OSNs in seven-, 30-, and 90-day-old *Adcy3*^-/-^ mice and wild-type controls (*Adcy*3^+/+^), we observed that the absence of *Adcy3* leads to cumulative defects in the maturation of OSNs. Upon aging, *Adcy3*^-/-^ OSNs exhibited increase in immature cells and reduction in mature cells along with elevated apoptosis levels. The density and ultrastructure of *Adcy3*^-/-^ cilia were also disrupted in mice upon aging. Collectively, our results reveal an indispensable role of Adcy3 in postnatal maturation of OSNs and maintenance of olfactory cilium ultrastructure in mice through adulthood.

## Introduction

The main olfactory epithelium (MOE) is a pseudostratified epithelial structure required for odor perception in mammals (Beites et al., 2005). The neural stem cells located in the basal epithelium give rise to olfactory sensory neurons (OSNs) (Jang et al., 2014). Several million OSNs, consisting of immature olfactory sensory neurons (iOSNs) and mature olfactory sensory neurons (mOSNs), are situated in the middle of the MOE. mOSNs are equipped with cilia, in which the olfactory cyclic adenosine monophosphate (cAMP) signaling cascade members, which are critical for proper olfactory function, are enriched (Menco, 1997; Heron et al., 2013). One unique characteristic of OSNs is their continuous neurogenesis that occurs throughout life. OSNs undergo caspase-mediated apoptosis at all stages of their life cycle (Mahalik, 1996). These cells are subsequently replenished by newly generated OSNs from the division of the basal stem cells to maintain epithelial homeostasis.

The equilibrium between OSN survival and apoptosis is possibly regulated by odorant-stimulated neural activity and sensory experience (Zhao et al., 2013; Cadiou et al., 2014; Kikuta et al., 2015). Sensory input plays a critical role in the survival of OSNs during postnatal MOE development (Farbman et al., 1988; Stahl et al., 1990; Brunjes, 1994; Coppola et al., 2006). Olfactory sensory deprivation in neonatal mice using unilateral naris occlusion results in a thinner MOE, fewer OSNs, and reduced number of olfactory marker protein (OMP)-positive cells (Coppola et al., 2006; Coppola, 2012).

The lifespan of OSNs was observed to be significantly longer in *H2BE* (an olfactory-specific histone variant) knockouts and shorter in *H2BE*-overexpressing mice (Santoro and Dulac, 2012). cAMP mediated the activity-dependent downregulation of H2BE (Santoro and Dulac, 2012). In addition, the activity-dependent survival of OSNs was promoted by cAMP pathway-dependent MAPK activation (Watt et al., 2004), implying that olfactory cAMP signaling might be required for the survival of OSNs in mice.

Interestingly, studies showed that the gross and microscopic anatomy of MOE in cAMP-signaling-cascade-member-knockout mice, including type 3 adenylyl cyclase (*Adcy3*), G-protein-olfactory-subunit (*G_olf_*), and cyclic-nucleotide-gated-channel α subunit (*CNGα*) knockouts, were indistinguishable from their wild-type littermates (Brunet et al., 1996; Belluscio et al., 1998; Wong et al., 2000). The similar appearance raised the possibility that odorant detection and stimulation-mediated OSN survival might be processed via independent signaling pathways (Kim et al., 2015). However, a limitation of these studies involved using 1-day-old pups as cAMP-cascade-component knockout mice. At this early age, architecture and associated composition of MOE are relatively underdeveloped. Accordingly, the full effects of sensory deprivation on maturation of OSNs may not be exhibited. For example, the MOE of OMP-*dnRAR* knockout and littermate control mice were indistinguishable in relation to cellular organization, thickness, and OMP immunoreactivity at postnatal day (P) 1, whereas both thickness of the MOE and OMP immunoreactivity were reduced in transgenic mice compared with littermates after 1 month (Hagglund et al., 2006). Moreover, cilium density and morphology were normal before P5 in Centrin2 (*CETN2*) knockout mice, whereas increased cilia loss was exhibited in P14 (and older) *CETN2* mutant MOE (Ying et al., 2014). The OSNs of *Adcy3*^-/-^ mice are devoid of odorant-evoked activity at both behavioral and electrophysiological levels (Wong et al., 2000). In addition, *Adcy3* expression in the OSNs of postnatal animals are both age- and location-dependent (Zou et al., 2007; Challis et al., 2015; Login et al., 2015), and the time of *Adcy3* expression significantly influences the behavior of OSN axons (Rodriguez-Gil et al., 2015). Accordingly, the absence of sensory input in *Adcy3*^-/-^ mice may lead to developmental impairment of postnatal OSNs upon aging.

In this study, we examined the OSNs of *Adcy3*^-/-^ mice and *Adcy3*^+/+^ littermates after seven, 30, and 90 postnatal days and demonstrated that the absence of *Adcy3* induced the formation of thinner MOE and increased loss of mOSNs along with elevated levels of apoptosis. Additionally, cilium density and ultrastructure were also severely disrupted in OSNs of *Adcy3*^-/-^ MOE.

## Materials and Methods

### Mice

*Adcy3*^+/-^ mice were obtained from the Storm Laboratory, University of Washington, Seattle, United States. *Adcy3*^+/+^ and *Adcy3*^-/-^ mice were bred from heterozygotes and genotyped using polymerase chain reaction (PCR), as previously described (Wang and Storm, 2006). All mice were housed under a 12 h light/dark cycle and provided with access to food and water *ad libitum* in a specific pathogen-free animal room in Hebei University. All experimental procedures were performed under the Guiding Opinions on the Treatment of Experimental Animals issued by the Ministry of Science and Technology, People’s Republic of China and approved by the Animal Ethics and Caring Committee of Hebei University.

### RNA Isolation and Quantitative Real-Time PCR

Main olfactory epitheliums from *Adcy3*^+/+^ (*n* = 3) and *Adcy3*^-/-^ (*n* = 3) mice at P7, P30, and P90 were dissected. RNA of MOE was isolated using traditional TRIzol method (Ambion, cat. nos. 208054). cDNA was synthesized using PrimeScript^TM^ RT Reagent Kit with gDNA Eraser (TaKaRa, cat. nos. RR047A). Quantitative *Adcy3* expression was determined through three-step quantitative reverse transcription polymerase chain reaction (qRT-PCR) using QuantiNava^TM^ SYBR Green PCR Kit (QIAGEN, cat. nos. 208054) (sequences of *Adcy3* primers are as follows: *Adcy3* F, AGATGTTCGGTGCCACCTG; *Adcy3* R, ACTTCACCAGGGCTTCGTAAG). *Adcy3* mRNA expression was determined using the 2^-ΔΔCT^ method (Livak and Schmittgen, 2001) and normalized to that of the housekeeping gene, *β-actin* [sequences of *β-actin* primers are as follows: *β-actin* F, CTAAGGCCAACCGTGAAAAG; *β-actin* R, ACCAGAGGCATACAGGGACA (Sciacovelli et al., 2016)].

### Processing of Samples and Tissue Sections for Light Microscopy

Mice were anesthetized with intraperitoneal injection of pentobarbital sodium (100 mg/kg body weight). Animals were transcardially perfused with 0.9% physiological saline followed by 4% paraformaldehyde (PFA, pH 7.4). The nasal cavity was postfixed overnight in 4% PFA at 4°C and was subsequently transferred into 10, 20, and 30% sucrose in phosphate-buffered saline (PBS) until sinking (older mice were decalcified in 1 M Ethylenediaminetetraacetic acid (EDTA), pH 7.4, at 4°C). Postfixed specimens were then embedded under optimal cutting temperature. The olfactory tissue was coronally sectioned using a freezing microtome (Leica 1950). The sections were used for either hematoxylin–eosin (HE) staining or immunofluorescence staining procedures.

### Scanning Electron Microscopy (SEM)

For SEM observations, *Adcy3*^-/-^ and *Adcy3*^+/+^ male mice (*n* = 3 each) aged P7, P30, and P90 were transcardially perfused with 2.5% glutaraldehyde (in 0.1 M phosphate buffer, pH 7.4). After fixation, the heads were hemisected, the nasal cavity was exposed under a dissecting microscope, and olfactory mucosa was removed from the dorsal zone along the medial aspect (where robust location-dependent change in cilium length was observed) and further fixed overnight in 2.5% glutaraldehyde at 4°C. The samples were washed four times with 0.1 M phosphate buffer (pH 7.4), dehydrated in increasing ethanol concentrations (50, 70, 80, 90, and 100%), and dried using a vacuum pump. The dried samples were then coated with gold particles (EIKO IB-3) and examined using the scanning microscope (Hitachi S-3500N) at 20 KV.

### Transmission Electron Microscopy (TEM)

For TEM observations, similar regions of the olfactory mucosa observed via SEM were examined using TEM in both *Adcy3*^-/-^ and *Adcy3*^+/+^ male mice (*n* = 3 each). The olfactory mucosa from mice aged P30 and P90 was fixed for 4 h in 4% glutaraldehyde (in 0.1 M phosphate buffer, pH 7.4) at 4°C. The samples were washed and postfixed for 2 h in 1% osmium tetroxide (pH 7.4) at 4°C. Washed specimens were dehydrated in increasing ethanol concentrations (50, 70, 80, 90, and 100%). The specimens were then dried in propylene oxide and infiltrated overnight with an Epon resin mix/propylene oxide (1:1) mixture. This step was followed by infiltration with 100% Epon resin for 48 h. The specimens were embedded and cured in plastic following incubation in an oven at 60°C for 48 h. The sections (thickness = 1 μm) were cut using an ultramicrotome (Lecia UC-7) until the desired region was reached. Ultrathin sections (50 nm thick) were then prepared, stained with uranyl acetate and lead citrate, and examined using TEM (Hitachi H-7000).

### Immunofluorescence

Immunofluorescence staining of tissue sections was performed according to a previously published protocol (Wang et al., 2011). The sections were rinsed with PBS (pH 7.4) and incubated in a blocking solution (containing 10% serum, 5% BSA, and 0.2% Tritonx-100) for 1 h at 37°C. The sections were then incubated with the following primary antibodies at 4°C overnight: goat anti-OMP (1:500; Wako, cat. nos. 019-22291), rabbit anti-growth associated protein-43 (GAP43) (1:500; Millipore, cat. nos. AB5220), mouse anti-acetylated (Ac)-α-tubulin (1:1000; Sigma–Aldrich, cat. nos. T6199), rabbit anti-cleaved caspase3 (1:200; cell signaling, cat. nos. 9661S), mouse anti-bromodeoxyuridine (BrdU, 1:200; Sigma–Aldrich, cat. nos. B5002), mouse anti-Ki67 (1:300; BD, cat. nos. 556003), and mouse anti-Mash1 (1:150; BD, cat. nos. AF796). After washing thrice with Phosphate Buffer Solution +Tween-20 (PBST), sections were incubated with reciprocal Alexa Fluor dye-conjugated secondary antibodies (1:500, Invitrogen) for 1 h at room temperature. The signal in Ki67 and Mash1-staining was amplified by the deposition of fluorescein using the TSA Fluorescein System (1:50; PerkinElmer Life, cat. nos. NEL701A001KT). The sections were counterstained with 4′6-diamidino-2-phenylindole (DAPI) (2 μg/mL; Sigma, cat. nos. D9542) and coverslipped with PBS, which instead of being used as primary antibody, was used in negative controls.

### BrdU Injections

To examine cell proliferations in MOE, male mice (*n* = 3 each group) aged P7 and P30 were intraperitoneally injected with BrdU (Sigma–Aldrich, cat. nos. B8434) (100 mg/kg body weight, dissolved in sterile saline) 2 h prior to perfusion. Male mice (*n* = 3 each group) aged P7, P30, and P90 were also injected thrice intraperitoneally (100 mg/kg body weight) (with intervals of 2 h) for three consecutive days. Mice were perfused 4 weeks later, and lifespan of neurons in the MOE was examined. PFA-fixed sections were incubated with 2 N HCl for 30 min at 37°C before immunostaining with anti-BrdU antibody. The subsequent immunostaining procedure was identical to that used for other antibodies.

### Imaging and Quantitative Analysis

HE-stained sections were examined and photographed using × 40 lens (NA, 0.95) of an Olympus BX53 under bright-field illumination. Immunofluorescence-stained sections were imaged using Olympus IX81 FLUOVIEW confocal microscope. Z-stack images (1024 × 1024 pixels) were captured using ×20 (NA, 0.75) and ×40 (NA, 0.95) objective lenses. In these image stacks, all labeled cells were counted, and we simultaneously determined whether these cells were double-labeled for OMP and caspase3. All images were only processed to facilitate contrast, brightness, and color-balance optimization using Adobe Photoshop CS software.

The thickness of MOE in HE sections, defined as the length from the basement membrane to the top of the knobs, was measured as documented in literature (Weiler and Farbman, 1997; Barrios et al., 2014). The same threshold was applied for all images obtained from the experiment. Laser intensity and detector gain were individually adjusted for each filter for maximum dynamic range, and settings were left unchanged for all the images collected in a section. In addition, no other attempts were made to normalize measurements background immunostaining. The background is a random factor, which is unrelated to the genotypes or treatments. Therefore, this measurement should lead to more conservative inferences. Positive cells under higher magnification images (40× objective) were manually counted using the “Cell Counter” tool of ImageJ plugins. For all quantifications, the experimenter was blinded to genotypes of the studied animals. The epithelial length along olfactory mucosa was measured using AxioVision software. Cell density was calculated by dividing the number of immunopositive cells by epithelial length (cells number/mm) for each age group. The number of immunopositive cells was finally converted based on the method published by Abercrombie (1946): corrected number = count × [section thickness/(section thickness + mean nucleus size)] (Abercrombie, 1946).

### Statistical Analysis

All data were expressed as mean ± standard error, unless otherwise indicated. Statistical analyses were performed with SPSS 21.0. Normality of distribution of variables and homogeneity of variances were checked through Shapiro–Wilk’s and Levene’s tests, respectively. We analyzed the data using one- or two-way ANOVA with *post hoc* pairwise comparisons. *p* < 0.05 was considered statistically significant.

## Results

### Deletion of *Adcy3* Reduces Cell Number and Thickness of the MOE

**FIGURE 1:**
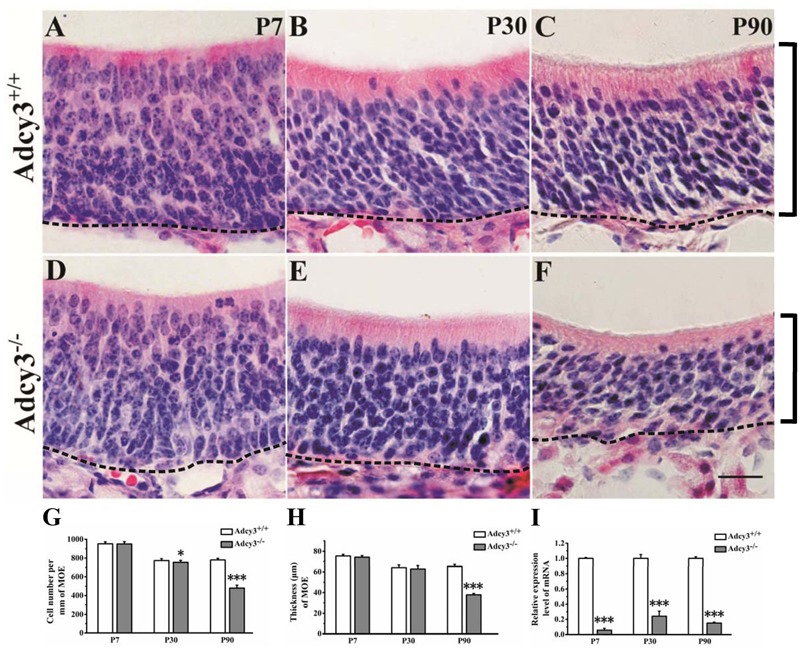
**Cell number and thickness of the MOE decreased in P90 *Adcy3*^-/-^ mice. (A–C)** Representative images of HE-stained sections of the MOE at P7 **(A)**, P30 **(B)**, and P90 **(C)** *Adcy3*^+/+^ mice. **(D–F)** Representative images of HE-stained sections from *Adcy3*^-/-^ mice at P7 **(D)**, P30 **(E)**, and P90 **(F)**. Cells were irregularly arranged in P90 *Adcy3*^-/-^ mice **(F)**. **(G)** Quantitative analysis of cell density of MOE, showing a slight reduction at P30 and substantial decrease in P90 *Adcy3*^-/-^ mice compared with that of *Adcy3*^+/+^ controls. **(H)** Quantitative analysis of MOE thickness, showing a substantial decrease in P90 *Adcy3*^-/-^ mice compared with of *Adcy3*^+/+^ controls. **(I)** Relative *Adcy3* mRNA expression levels in MOEs of P7, P30, and P90 mice. *Adcy3* expression level significantly decreased in *Adcy3*^-/-^ mice compared with that of *Adcy3*^+/+^ controls at all ages examined. Black brackets indicate thickness of MOE. Data are presented as mean ± standard error; *n* = 3 for each group; ^∗^*P* < 0.05, ^∗∗∗^*P* < 0.001. Scale bars: **(A–F)**, 20 μm.

By using qRT-PCR analysis, we confirmed that the *Adcy3* expression levels in MOEs of P7, P30, and P90 *Adcy3*^-/-^ mice are significantly lower compared with those of *Adcy3*^+/+^ mice with the same ages (**Figure 1I**). To evaluate whether the overall structure of the MOE is affected by *Adcy3* deletion, we performed HE staining on specimens from P7, P30, and P90 *Adcy3*^+/+^ and *Adcy3*^-/-^ mice. Our analyses were restricted to the mid-dorsal mucosa in each specimen to avoid morphological variations in the analyzed regions. The restriction was based on different regions of the MOE with different thicknesses. In general, the dorsal regions are thicker than the corresponding ventral regions, the medial regions are thicker than the lateral regions, and the middle and posterior regions are thicker than the anterior areas (Barrios et al., 2014).

In wild-type mice, results showed that the MOE at P7 was relatively thicker than those at P30 and P90, and the cells were loosely arranged (**Figure 1A**). Upon adulthood, the MOE became thinner, and neurons were more densely arranged (**Figures 1B,C**). These observations were consistent with those of previous histological studies, which showed the age-dependent decrease in epithelial thickness of murine MOE (Kondo et al., 2010). In *Adcy3*^-/-^ mice, the MOE cell density varied little from that of wild-type mice at P7 (*Adcy3*^-/-^, 949.36 ± 24.19/mm; *Adcy3*^+/+^, 951.07 ± 21.21/mm; *p* = 0.858; **Figures 1A,D,G**) and the MOE cell density at P30 was slightly reduced when compared with that of *Adcy3*^+/+^ mice (**Figures 1E,G**). However, in P90 *Adcy3*^-/-^ mice, the cell density of MOE sharply decreased (*Adcy3*^-/-^, 479.92 ± 31.67/mm; *Adcy3*^+/+^, 780.11 ± 17.05 /mm; *p* = 0.000; **Figures 1C,F,G**), and the MOE was much thinner (*Adcy3*^-/-^, 37.79 ± 1.34 μm; *Adcy3*^+/+^, 65.16 ± 1.95 μm; *p* = 0.000; **Figures 1C,F,H**) when compared with wild-type specimens. Compared with the cell density of *Adcy3*^+/+^ mice at P90, the cell density of MOE in *Adcy3*^-/-^ mice was 38.5% less at the same period of observation (**Figure 1G**). Together, these data suggest that *Adcy3* deletion results in the gradual decline in cell number after birth and profound reduction in adult mice. The complete structure of the MOE is apparently affected by *Adcy3* loss as animals age reach adulthood after the postnatal stages.

### Deletion of *Adcy3* Increases iOSN and Reduces mOSNs

**FIGURE 2:**
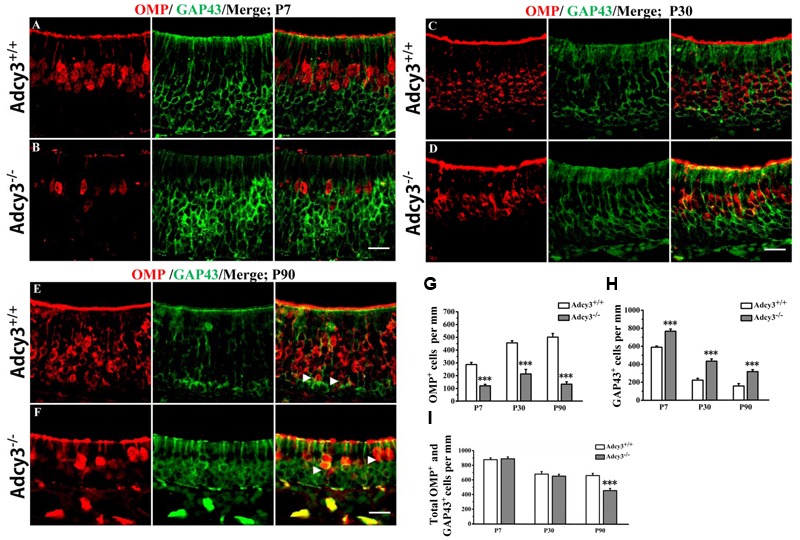
**Expression of OMP and GAP43 is altered in the MOE of *Adcy3*^-/-^ mice. (A–F)** Representative images of MOE sections stained with OMP and GAP43 (green) in *Adcy3*^+/+^ (top) and *Adcy3*^-/-^ (lower) mice at P7 **(A,B)**, P30 **(C,D)**, and P90 **(E,F)**. In *Adcy3*^+/+^ mice at ages from P7, P30, to P90, a reduction in GAP43^+^ cells and an increase in OMP^+^ cells were observed **(A,C,E)**. A distinct boundary was noted between OMP^+^ mOSN and GAP43^+^ iOSN in P90 *Adcy3*^+/+^ mice (**E**, arrowheads). GAP43 expression was more elevated in *Adcy3*^-/-^ mice compared with that of *Adcy3*^+/+^ controls, whereas OMP expression was substantially reduced **(B,D,F)**. The boundary between OMP and GAP43 was obscured in P90 *Adcy3*^-/-^ mice (**F**, arrowheads). **(G,H)** Quantitative analysis of OMP^+^ and GAP43^+^ cell numbers in *Adcy3*^+/+^ and *Adcy3*^-/-^ mice at all ages examined. **(I)** Quantitative analysis of the total OMP^+^ and GAP43^+^ cells in *Adcy3*^+/+^ and *Adcy3*^-/-^ mice at all ages examined. Data are presented as mean ± standard error; *n* = 3 for each group; ^∗∗∗^*P* < 0.001. Scale bars: (**A–F)**, 20 μm.

To further characterize OSN maturation in *Adcy3*^-/-^ mice, we examined the expressions of the immature OSN marker, GAP43, and the mature OSN marker, OMP, in the MOE. In *Adcy3*^+/+^ mice, the number of GAP43^+^ cells decreased from age P7 (589.25 ± 13.60/mm) and P30 (222.55 ± 20.10/mm) until P90 (157.49 ± 26.91/mm) (**Figures 2A,C,E,H**). Concurrently, an increase in OMP^+^ cells was observed following MOE development from age P7 and P30 until P90 (**Figures 2A,C,E,G**). By contrast, the *Adcy3*^-/-^ MOE exhibited a higher number of GAP43^+^ cells and significantly reduced OMP^+^ cells at all postnatal ages examined (**Figures 2B,D,F,G,H**). GAP43 expression was apically increased (*Adcy3*^-/-^, 319.23 ± 21.69/mm; *Adcy3*^+/+^, 157.49 ± 26.91/mm; *p* = 0.000) (**Figures 2E,F,H**), mOSN layer was reduced and mainly restricted to the most apical layer of the MOE, below the supporting cells in the MOEs of P90 *Adcy3*^-/-^ mice (*Adcy3*^-/-^, 134.38 ± 17.79/mm; *Adcy3*^+/+^, 501.59 ± 28.43/mm; *p* = 0.000; **Figures 2E,F,G**). Remarkably, the number of OMP^+^ cells in P90 *Adcy3*^-/-^ mice was much less than that observed in P30 *Adcy3*^-/-^ mice (P30, 213.46 ± 35.52/mm; P90, 134.38 ± 17.79/mm; *p* = 0.000; **Figures 2D,F,G**), which is obviously not consistent with normal OSN development patterns exhibited in *Adcy3*^+/+^ mice. Although the total OSN number (OMP^+^ and GAP43^+^) between *Adcy3*^+/+^ and *Adcy3*^-/-^ mice varied little at P7 and P30, it significantly decreased in *Adcy3*^-/-^ mice at P90 (**Figure 2I**). Together, these data demonstrate that *Adcy3* deletion reduces mOSN number and inhibits OSN development at the immature neuronal stage of mice during adulthood.

### Deletion of *Adcy3* Reduces the Lifespan of OSNs

**FIGURE 3:**
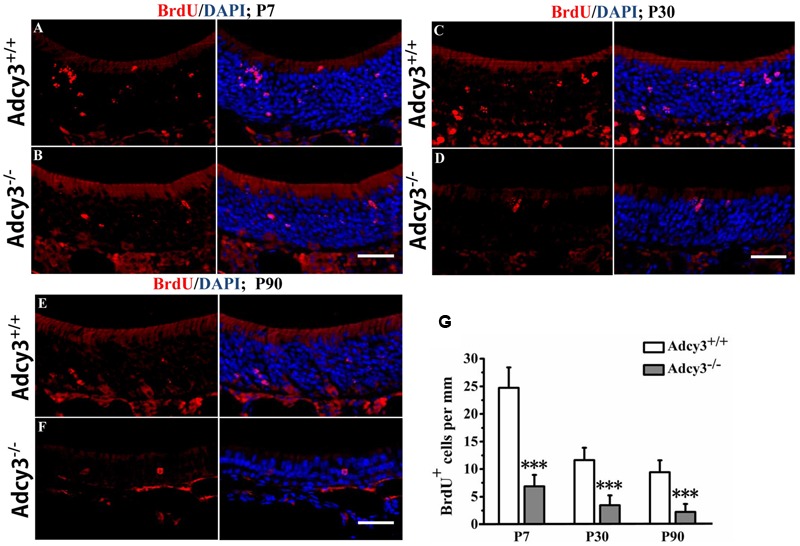
**Olfactory sensory neuron (OSN) lifespan is shortened in postnatal *Adcy3*^-/-^ OSNs. (A–F)** Representative images of the MOE sections stained with BrdU and nuclear DAPI (blue) in P7 **(A,B)**, P30 **(C,D)**, and P90 **(E,F)** *Adcy3*^+/+^ and *Adcy3*^-/-^ mice; 4 weeks post-BrdU injection. Compared with *Adcy3*^+/+^ mice, *Adcy3*^-/-^ mice showed significantly fewer BrdU^+^ cells at all ages examined. **(G)** Quantification of BrdU^+^ cells in the MOE of *Adcy3*^+/+^ and *Adcy3*^-/-^ mice 4 weeks post-BrdU injection. Data presented as mean ± standard error; *n* = 3 for each group; ^∗∗∗^*P* < 0.001. Scale bars: **(A–F)**, 40 μm.

One hypothesis regarding the reduced cell numbers in the OSNs is that this phenomenon arises from reduction in cell lifespan, increase in apoptotic cell number, and/or decreased proliferation of progenitors. To test this possibility, we utilized long-term BrdU incorporation analysis to measure the OSN lifespan. *Adcy3*^-/-^ mice and wild-type littermates were injected with BrdU at ages P7, P30, and P90. Mice were subsequently sacrificed 4 weeks after injection. The number of BrdU^+^ cells in *Adcy3*^-/-^ mice significantly decreased relative to *Adcy3*^+/+^ controls at each of the examined stage (**Figures 3A–G**). These data indicate that *Adcy3* deletion shortens the lifespan of OSNs.

### *Adcy3* Deletion Does Not Suppress Cell Proliferation in MOE

**FIGURE 4:**
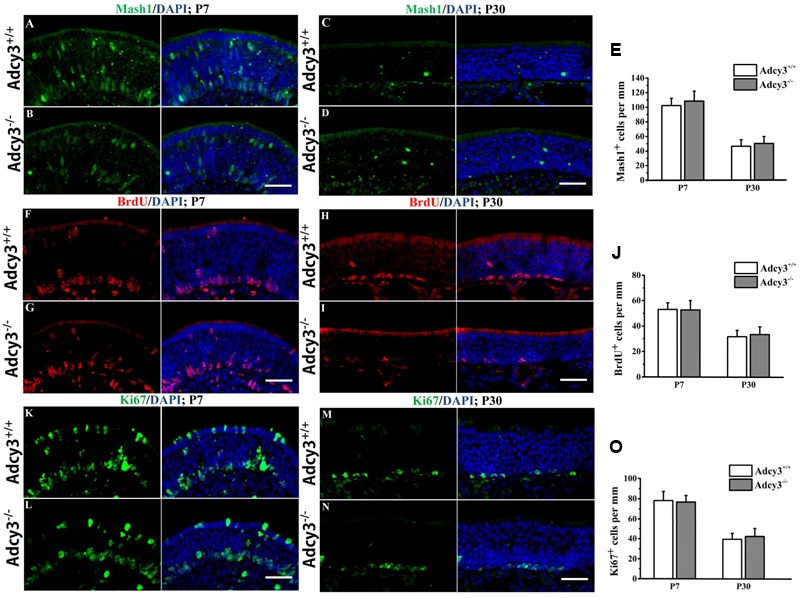
**Cell proliferation is normal in the *Adcy3*^-/-^ MOE. (A–D)** Representative images of MOE sections stained with Mash1 (green) and nuclear DAPI (blue) in *Adcy3*^+/+^ and *Adcy3*^-/-^ mice at P7 **(A,B)** and P30 **(C,D)**. Mash1^+^ cells are expressed in the apical, intermediate, and basal regions of the MOE at P7 **(A,B)**. Mash1^+^ cells are significantly reduced at P30 **(C,D)** in both *Adcy3*^+/+^ and *Adcy3*^-/-^ mice. No significant difference exists between *Adcy3*^+/+^ and *Adcy3*^-/-^ mice at P7 and P30. **(F–I)** Representative images of MOE sections stained with BrdU and nuclear DAPI (blue) in A*dcy3*^+/+^ and *Adcy3*^-/-^ mice at P7 **(F,G)** and P30 **(H,I)**. Male mice were sacrificed 2 h after BrdU injection. We observed increased progenitors in the apical and basal positions in both *Adcy3*^+/+^ and *Adcy3*^-/-^ mice at P7 **(F,G)** and decreased at P30 **(H,I)**. Difference was not observed between *Adcy3*^+/+^ and *Adcy*3^-/-^ MOE at these ages. **(K–N)** Representative images of MOE sections stained with Ki67^+^ (another marker for cell proliferation, green) cells in the MOE of *Adcy3*^+/+^ and *Adcy*3^-/-^ mice at P7 **(K,L)** and P30 **(M,N)**. Similar Ki67 expression levels were observed between *Adcy3*^+/+^ and *Adcy3*^-/-^ mice at the two stages. **(E,J,O)** Quantitative analysis of Mash1^+^, BrdU^+^, and Ki67^+^ cells in the *Adcy3*^+/+^ and *Adcy3*^-/-^ MOE at P7 and P30. Data are shown as mean ± standard error; *n* = 3 for each group. Scale bars: (**A–D)**, **(F–I)**, and **(K–N)**, 40 μm.

To assess whether reduction of OSNs in *Adcy3*^-/-^ mice resulted from alterations to cell proliferation mechanisms, we analyzed the expression of the neuronal progenitor marker, Mash1, a marker for globose basal cells (GBCs). Mash1 expression did not differ between *Adcy3*^-/-^ and *Adcy3*^+/+^ mice at P7 and P30 (**Figures 4A–E**), indicating that OSN progenitors are possibly not affected by the loss of *Adcy3* and develop normally into neurons at the early stages of neurogenesis. We identified actively proliferating cells using two markers, BrdU and Ki67, which are both used as labels for multiplying cells. Animals were injected with a single dose of BrdU and perfused after 2 h. The MOE sections were immunolabeled with antibodies against BrdU and Ki67. At P7, *Adcy3*^+/+^ and *Adcy3*^-/-^ MOE showed a substantial number of BrdU-labeled proliferating cells (**Figures 4F,G**). BrdU^+^ cells were predominantly scattered near the basal neuroepithelium at both genotypes of P30 mice (**Figures 4H,I**). Furthermore, BrdU^+^ cell numbers also decreased with aging in *Adcy3*^+/+^ and *Adcy3*^-/-^ mice (**Figure 4J**). However, significant difference was not observed between *Adcy3*^+/+^ and *Adcy3*^-/-^ mice at P7 and P30 in terms of BrdU^+^ cell numbers (**Figure 4J**). Ki67^+^ cells were expressed in both the basal and apical layers of MOEs in the *Adcy3*^+/+^ and *Adcy3*^-/-^ mice at P7 (**Figures 4 K,L**), and were mainly restricted to the basal layer at P30 (**Figures 4M,N**). Compared with P7 mice, the Ki67^+^ cells decreased at P30 in both *Adcy3*^+/+^ and *Adcy3*^-/-^ MOEs (**Figure 4O**). Similarly, the number of Ki67^+^ proliferating cells varied minimally between *Adcy3*^-/-^ and *Adcy3*^+/+^ mice at P7 and P30 (**Figure 4O**). Together, these data indicate that *Adcy3* deletion does not suppress cell proliferation in MOE.

### Deletion of *Adcy3* Increases Apoptotic Cell Number of mOSNs

**FIGURE 5:**
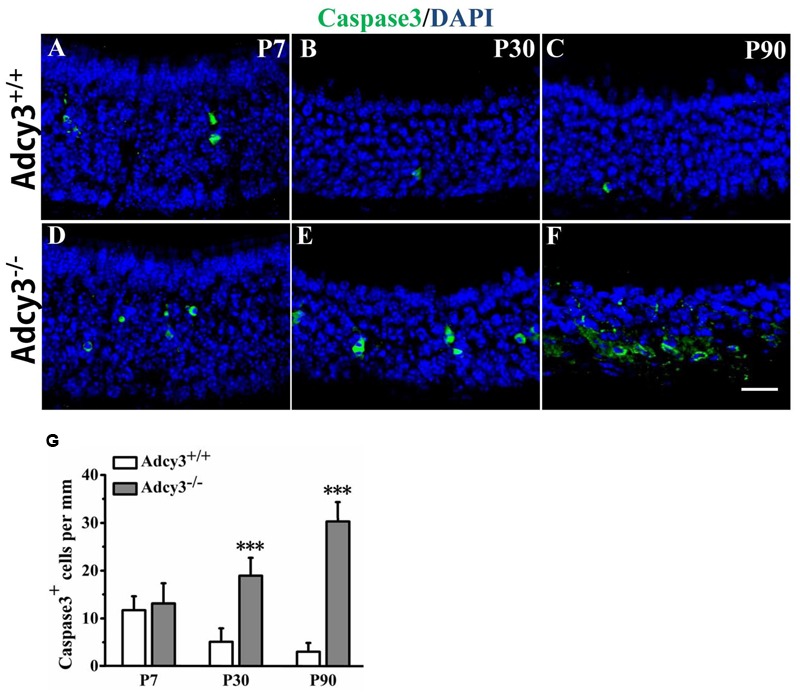
**Increased apoptosis in *Adcy3*^-/-^ MOEs. (A–F)** Representative images of the MOE sections stained with cleaved caspase3 (green) and nuclear DAPI (blue) in P7 **(A,D)**, P30 **(B,E)**, and P90 **(C,F)** *Adcy3*^+/+^ and *Adcy3*^-/-^ mice. In *Adcy3*^+/+^ mice, the number of caspase3^+^ cells declined with increasing age **(A,B,C)**. Limited numbers of apoptotic cells are restricted to the basal epithelium in P30 and P90 *Adcy3*^+/+^ MOE **(B,C)**. *Adcy3*^-/-^ mice at P30 and P90 displayed increased apoptotic cells in comparison with wild-type mice **(E,F)**. **(G)** Quantification of caspase3^+^ cells in the MOE of *Adcy3*^+/+^ and *Adcy3*^-/-^ mice at P7, P30, and P90. Data are shown as mean ± standard error; *n* = 3 for each group; ^∗∗∗^*P* < 0.001. Scale bars: **(A–F)**, 20 μm.

To determine whether the decrease in OSN in *Adcy3*^-/-^ MOE resulted from an overall increase in postnatal apoptosis, we tested for the expression of cleaved-caspase3, an enzyme critically involved in mammalian apoptotic pathway. Compared with P7 *Adcy3*^+/+^ MOE, the number of caspase3^+^ cells decreased at P30 and P90 (**Figure 5G**). Only relatively few apoptotic cells were observed in the basal layer of MOE at P30 and P90 *Adcy3*^+/+^ mice (**Figures 5B,C,G**). The number of caspase3^+^ cells differed minutely between *Adcy3*^-/-^ and *Adcy3*^+/+^ mice at P7 (**Figures 5A,D,G**). However, we observed a significant increase in the number of caspase3^+^ cells in P30 and P90 *Adcy3*^-/-^ mice compared with their *Adcy3*^+/+^ controls (**Figures 5B,C,E,F,G**). The number of apoptotic cells in P90 *Adcy3*^-/-^ mice was approximately 10-fold higher than that observed for *Adcy3*^+/+^ littermates (**Figure 5G**). These results demonstrate that *Adcy3* deletion leads to pronounced elevation in apoptotic OSNs during MOE development.

**FIGURE 6:**
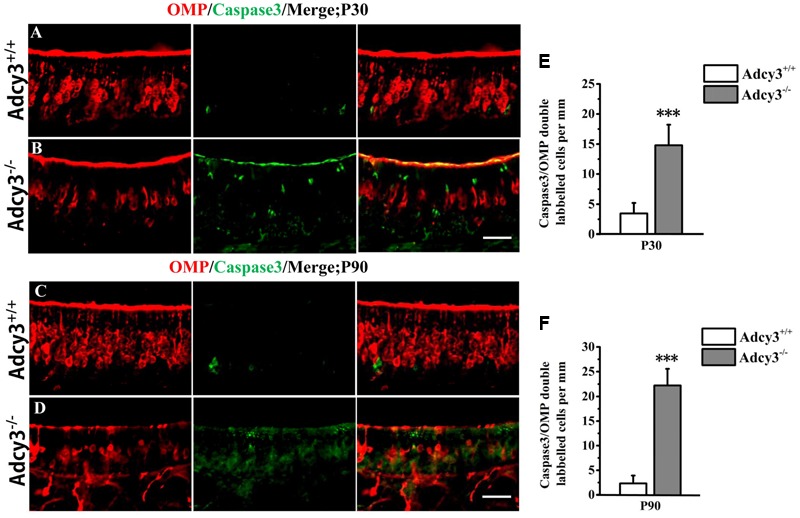
**Survival of mOSNs is impaired in *Adcy3*^-/-^ MOE. (A–D)** Representative images of MOE sections stained with cleaved caspase3 (green) and OMP (red) in P30 **(A,B)** and P90 **(C,D)** *Adcy3*^+/+^ and *Adcy3*^-/-^ mice. In *Adcy3*^+/+^ mice, some double-labeled cells were observed **(A,C)**. At P30 and P90, *Adcy3*^-/-^ mice displayed higher OMP and caspase3 double-labeled cells compared with that of wild-type mice **(B,D)**. **(E,F)** Quantitative analysis of OMP and caspase3 double-labeled cells in MOE of *Adcy3*^+/+^ and *Adcy3*^-/-^ mice at P30 and P90. Data are shown as mean ± standard error of mean; *n* = 3 mice for each group; ^∗∗∗^*P* < 0.001. Scale bars: (**A–D)**, 40 μm.

Although the total OSN is almost the same between *Adcy3*^+/+^ and *Adcy3*^-/-^ mice at P7 and P30 (**Figure 2I**), the total OSN substantially decreased in *Adcy3*^-/-^ mice at P90 (**Figure 2I**), and this reduction is mainly caused by the decrease in mOSN (**Figure 2G,H**). We performed double-staining of OMP and caspase3 in P30 and P90 MOE to determine whether apoptosis caused the mOSN reduction in *Adcy3*^-/-^ mice. Some OMP and caspase3 double-labeled cells were observed in *Adcy3*^+/+^ mice at both P30 and P90 (**Figures 6A,C,E,F**). Compared with *Adcy3*^+/+^ mice, the number of double-labeled mOSN cells significantly increased in *Adcy3*^-/-^ mice at both P30 and P90 (**Figures 6B,D,E,F**). These data suggest that the impairment of OSN survival may account for the decreased mOSN in *Adcy3*^-/-^ mice.

### Deletion of *Adcy3* Causes Loss of Olfactory Cilia and Disrupts Cilium Ultrastructure

Olfactory cilia are essential organelles that permit the conversion of external chemical stimuli into intracellular electrical responses in OSNs (Menco, 1997). Cilia stem from each dendritic knob of OSN and run horizontally in various directions to form a meshwork with cilia from other OSNs. This intertwined mat of cilia substantially increases the sensory surface of OSNs to facilitate odorant detection. In this study, aging culminated in a marked decrease of mOSN in *Adcy3*^-/-^ mice. To investigate whether cilia morphology was also altered by loss of mOSN after *Adcy3* deletion, we performed an Ac-α-tubulin (a ubiquitous cilium marker) immunofluorescence analysis and SEM.

**FIGURE 7:**
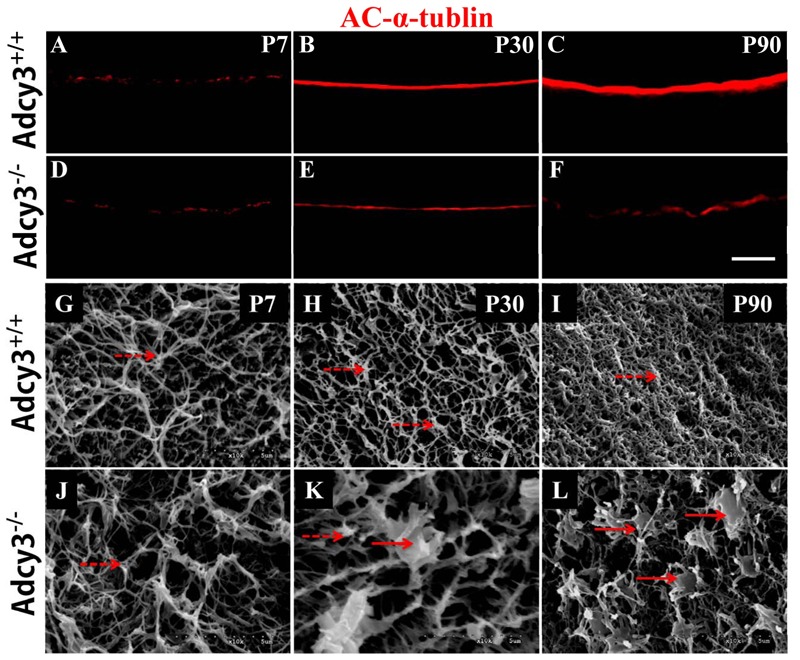
**Loss of olfactory cilia and disrupted cilium ultrastructure were observed in OSNs of *Adcy3*^-/-^ mice. (A–F)** Representative images of AC-α-tubulin immunolabeling in *Adcy3*^+/+^ **(A–C)** and *Adcy3*^-/-^ **(D–F)** mice at all ages examined. In *Adcy3*^+/+^ mice, the intensity of AC-α-tubulin-labeled ciliary layer becomes heavier with increasing age **(A–C)**. A significant decrease in the intensity of ciliary layer is observed in P30 and P90 *Adcy3*^-/-^ mice **(E,F)**. **(G–L)** SEM images of *Adcy3*^+/+^ **(G–I)** and *Adcy3*^-/-^ **(J–L)** mice at P7, P30, and P90. Cilia run horizontally in various directions to form increasingly dense meshworks in the epithelial surface of *Adcy3*^+/+^ mice from P7 **(G)** and P30 **(H)** until P90 **(I)**. *Adcy3*^-/-^ mice at P30 and P90 showed significant decrease in cilium density **(K,L)**. Both normal (dashed arrows) and abnormal dendritic knobs (solid arrows) were identified in P30 *Adcy3*^-/-^ mice **(K)**. Moreover, the number of enlarged knobs with short and stubby cilia increased in P90 *Adcy3*^-/-^ mice **(L)**. Scale bars: **(A–F)**, 20 μm; **(G–L)**, 5 μm.

Ac-α-tubulin immunolabeling revealed a substantial decrease in immunofluorescence intensity of the ciliary layer in *Adcy3*^-/-^ mice at P30 and P90 (**Figures 7A–F**). Following SEM analysis of wild-type specimens at P7, P30, and P90, olfactory cilia were observed to be oriented parallel to the epithelial surface. The structures were also observed to run in various directions, forming a fine and dense meshwork with increasing age (**Figures 7G–I**). This effect occurred in parallel with an increase in mOSN number of postnatal MOE. Substantial and dense overlaps were observed in the cilia of P90 *Adcy3*^+/+^ mice (**Figure 7I**). In *Adcy3*^-/-^ mice, cilia density was normal during early postnatal stages (P7, **Figure 7J**) but was significantly reduced at P30 and P90 (**Figures 7K,L**). Additionally, malformed dendritic knobs were observed in older specimens (especially P90). Numerous *Adcy3*^-/-^ cilia were short and stubby with enlarged knobs (**Figures 7K,L**, solid arrows). These results revealed that the loss of olfactory cilia occurs in conjunction with decreasing mOSNs in *Adcy3*^-/-^ mice.

**FIGURE 8:**
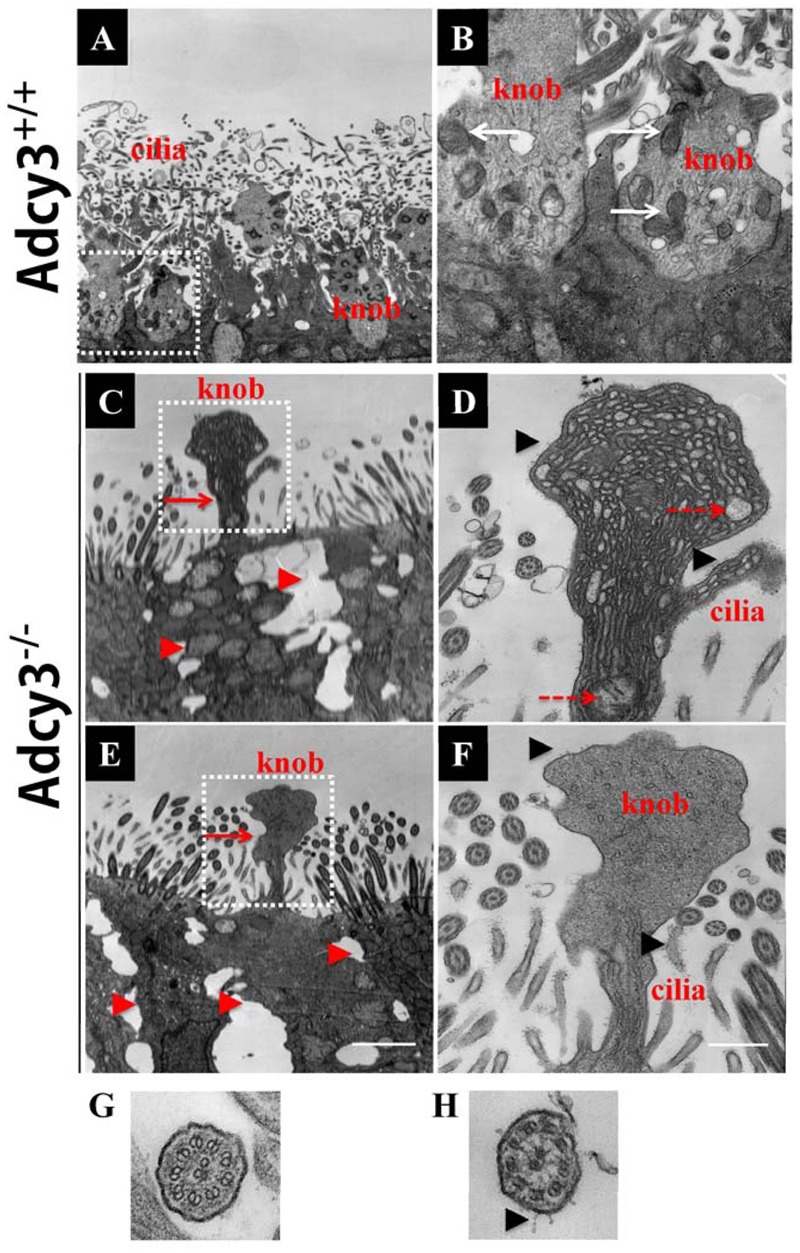
**Investigating disruption of cilium ultrastructure in P30 *Adcy3*^-/-^ OSNs using TEM. (A,B,G)** Cilium images in *Adcy3*^+/+^ mice. **(C–F,H)** Cilium images of *Adcy3*^-/-^ mice. **(A)** Normal olfactory cilia and dendritic knobs are present in *Adcy3*^+/+^ OSNs. (**B**) Increased magnification of the boxed area in **(A)** shows a substantial number of mitochondria (white arrows) in knobs and normal membranes (of cilia and knobs). **(C,E)** Malformed knobs (red arrows) and enlarged cell intervals (red arrowheads) are observed in *Adcy3*^-/-^ OSNs. **(D,F)** Increased magnification of the boxed areas in **(C)** and **(E)**. Membrane structure of cilia and knobs displays abnormal electron-dense areas (black arrowheads). Disrupted mitochondria is dissolved and fractured in some knobs **(D**, red dashed arrows). Some knobs lacked mitochondrial structures in **(F)**. **(G,H)** TEM cross view of cilia shows normal ciliary membranes and a “9 × 2” architecture in *Adcy3*^+/+^ mice **(G)**. Membranes displaying an electron-dense area of mutant cilia in *Adcy3*^-/-^ mice **(H)**. Scale bars: **(A,C,E)**, 2 μm; **(B,D,F)**, 667 nm; **(G,H)**, 100 nm.

**FIGURE 9:**
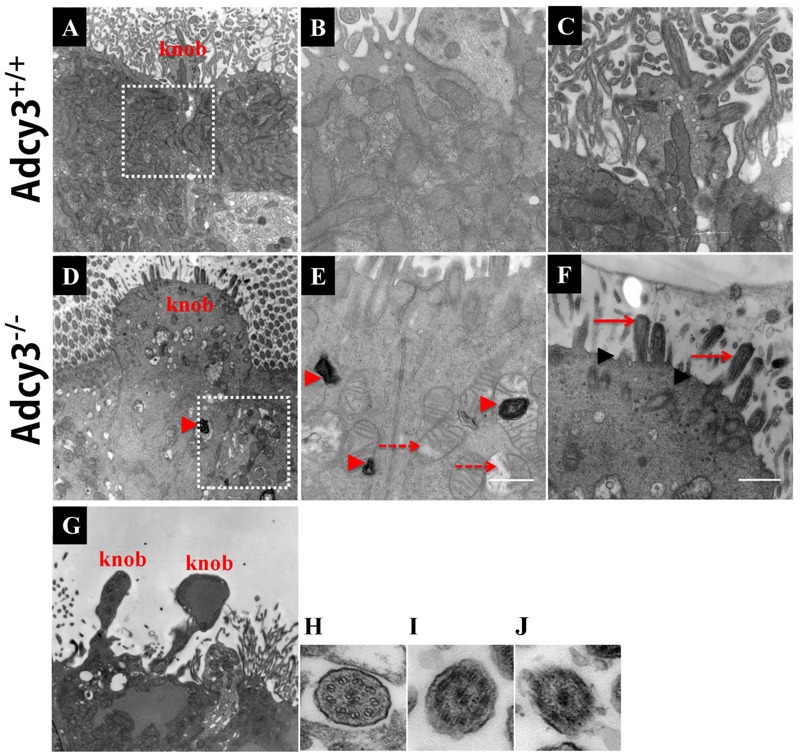
**Severely disrupted cilium ultrastructure in P90 *Adcy3*^-/-^ OSNs investigated using TEM. (A–C,H)** Cilium ultrastructure images of *Adcy3*^+/+^ mice. **(D–G,I)** Cilium ultrastructure images of *Adcy3*^-/-^ mice. **(A)** Normal olfactory cilia and dendritic knobs were observed in *Adcy3*^+/+^ OSNs. **(B)** Increased magnification of boxed area in **(A)** shows mitochondrial structures. **(C)** Increased magnification of the knob in **(A)** exhibits a complete membrane and numerous emanating cilia. **(D)** Enlarged knobs are observed in *Adcy3*^-/-^ OSNs. **(E)** Increased magnification of boxed area in **(D)** shows dissolved mitochondria (red dashed arrows) and medullary structures (red arrowheads). **(F)** Increased magnification of the knob in **(D)** displays an incomplete membrane structure (black arrowheads) and extensively swollen cilia (red solid arrow). **(G)** TEM of bare knobs in *Adcy3*^-/-^ OSNs illustrates lacking cilia and mitochondrial structures. **(H,I,J)** TEM cross section view of cilia in *Adcy*3^+/+^ **(H)** and *Adcy3*^-/-^ mice shows disintegrated cilium membrane **(I)** and disrupted ciliary “9 × 2” structure **(J)**. Scale bars: **(A,D,G)**, 2 μm; **(B,C,E,F)** 667 nm; **H,I,J** = 100 nm.

Given that reduced cilium density and abnormal morphology of dendritic knobs were observed at P30 and P90 in *Adcy3*^-/-^ mice, we further examined cilium ultrastructure via TEM. Ciliary microtubule configuration (9 + 2 arrangement) and dendritic knobs filled with mitochondria and numerous microtubules were observed in *Adcy3*^+/+^ mice at both P30 and P90 (**Figures 8A,B,G**; and **9A,B,C,H**). These results were consistent with those previously observed (Jenkins et al., 2009). The membrane structures of both cilia and knobs were normal in *Adcy3*^+/+^ mice. By contrast, the membranes displayed abnormal electron-dense outgrowths in P30 *Adcy3*^-/-^ mice (**Figures 8C,D,F,H**; black arrowheads). We frequently observed malformed knobs with dissolved and fractured mitochondria (**Figures 8C,D**, dashed arrows) and knobs lacking mitochondrial structures (**Figures 8E,F**). More severe ultrastructural deficiency was observed in P90 *Adcy3*^-/-^ mice. Many cilia and knobs had incomplete membranes (**Figures 9D,F**, black arrowheads, **Figure 9I,J**). *Adcy3*^-/-^ cilia were swollen (**Figures 9D,F**, red solid arrows), and some ciliary configurations (9 + 2) were disordered (**Figure 9J**). Medullary mitochondria were detected in the knobs (**Figures 9D,E**, red arrowheads), indicating severe mitochondrial ultrastructure damage. We also observed “bare knobs” with no cilia (**Figure 9G**). This phenomenon is rarely encountered in *Adcy3*^+/+^ mice. These findings suggest that *Adcy3*, which is a key ciliary membrane protein, plays a role in the maintenance of cilium ultrastructure during OSN development.

## Discussion

Although Adcy3 is required for odor perception (Wong et al., 2000; Wang et al., 2006) and OSN axonal projection formation (Trinh and Storm, 2003; Chesler et al., 2007; Col et al., 2007; Zou et al., 2007), its role in postnatal OSN maturation and cilium ultrastructure remains unclear. In this study, we showed that *Adcy3*^-/-^ mice exhibited cumulative disruptions in the overall structure of the MOE, OSN maturation, and cilium ultrastructure from postnatal to adult stages.

Each OSN selects an individual odor receptor gene to be expressed from a set of approximately 1200 olfactory receptor (OR) genes in a process called the OR choice. The OR choice identifies the detected odors and sends axons to particular targets in the main olfactory bulb (MOB). OR expression can be regulated by Adcy3 through a feedback loop via the downregulation of histone dimethyl enzyme LSD1; this enzyme locks in the singular choice of one allele of one OR gene (Lyons et al., 2013). Given that OR expression is a sign of OSN relative maturity, OSN differentiation is determined by OR choice and expression (Iwema and Schwob, 2003). OSNs with OR misexpression caused by disruptions in OR gene choice may be rendered uncompetitive and remain immature (Hanchate et al., 2015). This phenomenon implies the possibility that the OSN maturation retardation exhibited by the *Adcy3*^-/-^ mice in the present study is at least, in part, a consequence of a disruption in the regulation of OR gene choice and/or expression (Lyons et al., 2013). The mechanism of OR singular expression is extremely challenging and is possibly regulated on multiple axes (Fleischmann et al., 2013). Aside from epigenetic feedback loop regulation (Lyons et al., 2013), other signal pathways may also be involved in this process. These pathways include recently identified mechanisms that are independent of canonical G-protein signaling and cAMP production (Movahedi et al., 2016). Similarly, post-selection refinement may be involved, such that a competitive relationship between OR alleles could mediate post-selection shutdown (Abdus-Saboor et al., 2016). Given that Adcy3 is temporally and spatially expressed in the subcellular locations of the cilia and axons in OSNs of MOE (Maritan et al., 2009; Challis et al., 2015; Login et al., 2015; Rodriguez-Gil et al., 2015; Scholz et al., 2016), the dynamic timing and localization of Adcy3 expression (before, during, and after OR choice) may possibly play important roles in monogenic and monoallelic OR gene expression, OSN survival, and axon targeting. For example, *Adcy3*^-/-^ mice are anosmic because of the absence of Adcy3 expression in dendritic cilia (Wong et al., 2000). By contrast, glycosyltransferase *β3GnT2*^-/-^ mice are not anosmic because Adcy3 is normally trafficked to cilia (Knott et al., 2012). Both mice showed similar deficits in axon wiring and maturation impairments of OSNs because of the significantly reduced Adcy3 expression on the axon termini-growth cone (Henion et al., 2005, 2011; Knott et al., 2012). Transgenic OR gene expression using the OMP promoter exerts minimal effect on MOB architecture, whereas early induction of OR expression using the Gγ8 promoter, which is expressed in immature OSNs, alters the projection patterns of many residual OSNs (Nguyen et al., 2010). Furthermore, even OSNs expressing an identical OR represent a non-homogeneous population (Grosmaitre et al., 2006) and can express different levels or variants of *Adcy3* transcripts (Col et al., 2007). Accordingly, temporal and spatial *Adcy3* conditional knockouts in mice, combined with single-cell RNAseq (Hanchate et al., 2015; Tan et al., 2015; Scholz et al., 2016) on OSNs isolated from *Adcy3* knockout versus wild-type controls, may be required to completely determine whether OSN maturation retardation in the *Adcy3*^-/-^ mice in this study resulted from a disorder in OR gene choice.

Although our data showed that mOSNs notably decreased, iOSN significantly increased in *Adcy3*^-/-^ mice compared with their wild-type controls at all examined ages. The total OSN was not altered between *Adcy3*^+/+^ and *Adcy3*^-/-^ mice at P7 and P30, whereas it significantly decreased in *Adcy3*^-/-^ mice at P90. Given that OR gene switching primarily occurs at early developmental stages (Shykind et al., 2004), and disruption in OR gene choice may cause defects in age-independent OR expressions (Santoro and Dulac, 2012), then disruption in OR gene choice (Lyons et al., 2013) may not be the only cause of the maturation impairment of postnatal OSNs during adulthood in *Adcy3*^-/-^ mice. Considering that OR gene choice, OSN lifespan, and OSN survival are all influenced by odorant-stimulated neuronal activity and are cAMP signaling dependent (Zhao and Reed, 2001; Watt et al., 2004; Tian and Ma, 2008; Santoro and Dulac, 2012), and that OSNs of *Adcy3*^-/-^ mice are devoid of odorant-induced activity at both behavioral and electrophysiological levels (Wong et al., 2000), then the absence of sensory input in *Adcy3*^-/-^ mice may also account for the maturation impairment of postnatal OSNs in adult mice.

Interestingly, mice with overexpressed OAZ, the Olf/EBF-associated zinc finger protein in OSNs (O/E3 OAZ/^+^), demonstrated phenotypic copies of *Adcy3*^-/-^ mice. Both mice showed pleiotropic phenotypes of decreased mOSN, increased iOSNs, axon projection defects, altered OR gene choice, and increased apoptosis (Cheng and Reed, 2007; Roby et al., 2012). Given that OAZ is an inhibitor of O/E transcription factors preventing O/E transcriptional target expression, and that these transcription factors have putative binding sites in the promoter region of *Adcy3* gene (Robert and Tsai, 1997), these transcription molecules (Wang et al., 1993, 2012) and their repressor protein, ORZ, possibly play a role in OSN maturation through Adcy3 activation. Future studies should focus on details of mechanisms underlying OSN maturation.

In our study, the *Adcy3*^+/+^ MOE showed an age-dependent decrease in apoptosis, with a limited number of apoptotic cells observed in the basal epithelium of adult specimens. However, the MOE of *Adcy3*^-/-^ mice displayed high level of cell apoptosis, eventually leading to seriously atrophied and thin epithelia. Although the survival and maturation of newly generated granule cells (GCs) were shown to cause disturbance in adult *Adcy3*^-/-^ mice (Luo et al., 2015), MOB was suggested to promote a feedback mechanism that facilitates survival of OSNs in MOE (Schwob et al., 1992). The MOE, as the first relay station for olfactory information processing to the brain, sends information through OSN axons to the MOB. OSN neurogenesis occurs as a predominantly local phenomenon after birth in mice. Furthermore, axons arising from neurons expressing the P2 odorant receptor can still form glomerular-like loci following MOB removal (Bulfone et al., 1998; St John et al., 2003). In addition, regenerated olfactory axons demonstrated the capacity to form glomerular layers in the presence of an artificial biological scaffold, which is similar in size and shape to the MOB (Chehrehasa et al., 2006). Conversely, MOB development was shown to be closely associated with olfactory activity (Brunjes, 1994; Cummings and Brunjes, 1997; Petreanu and Alvarez-Buylla, 2002). Accordingly, we favor the hypothesis that the MOB, with regard to its role as mediator in processing of olfactory information from the MOE, undergoes developmental impairment in *Adcy3*^-/-^ mice (Luo et al., 2015) as a consequence of the absence of sensory input from the OSNs (Wong et al., 2000; Wang et al., 2006), rather than a contributor to the attenuated OSN maturation of *Adcy3*^-/-^ mice described here.

Olfactory sensory neurons in the MOE are generated by two types of basal stem cells: frequently dividing GBCs and dormant horizontal basal cells (HBCs). The possession of primary cilia is a unique characteristic of HBCs. Depletion of HBC cilia resulted in impaired regeneration of the OSNs owing to disrupted HBC proliferation (Joiner et al., 2015). Adcy3 is expressed in the primary cilia of HBCs. These data suggest that the impairment of OSN maturation observed in *Adcy3*^-/-^ mice might be caused by HBC primary cilia dysfunction. However, our data showed that MOE-specific cell proliferation proceeded normally in *Adcy3*^-/-^ mice. This observation rules out the possibility that disruption of OSN maturation in *Adcy3*^-/-^ mice is a consequence of defects in primary cilia structure/function of HBCs in the MOE.

Olfactory cilia are important organelles for OSNs in the conversion of external odor stimuli into intracellular electrical signals. Olfactory signaling cascades, which include major ORs, G_olf_, Adcy3, and CNG, are highly enriched in olfactory cilia (Brunet et al., 1996; Belluscio et al., 1998; Wong et al., 2000; Mombaerts, 2006). OSNs typically have short dendrites that extend apically and terminate in dendritic knobs at the epithelial surface. Cilia emanate from each knob of the OSN, with associated long and thin distal segments interacting with cilia from nearby knobs, forming a parallel meshwork. The expression patterns of cilia are positively correlated with odor perception competence. Damage to structure or function of associated cilia leads to olfactory dysfunction (Mukhopadhyay et al., 2008; Challis et al., 2015, 2016). As part of this study, we observed that the *Adcy3*^-/-^ cilium density was not significantly different from wild-type cilium density at P7. This indicates that Adcy3 might not affect the initiation of olfactory ciliogenesis. In the MOE, olfactory ciliogenesis is initiated at around embryonic day (E) 12 (Cuschieri and Bannister, 1975). However, ciliary signaling proteins are synthesized at a later stage. For example, in rats, mRNA expression of *Adcy3* is first detected at E15, whereas G_olf_ and CNGA2 are detectable from E16 and E19, respectively (Margalit and Lancet, 1993). Accordingly, the absence of *Adcy3* does not obviously influence cilium patterns at early developmental stages. However, aging leads to disruption in both the density and ultrastructural morphology of the cilia in *Adcy3*^-/-^ mice. This phenomenon included the disruption of membrane structures of OSNs, resulting in the retardation of the typical “9 + 2” ciliary architecture and bare knobs. The cumulative disruptions that appeared in cilia were consistent with the increasing number of apoptotic mOSN in postnatal developmental of MOE after *Adcy3* deletion.

Centrins are classic calmodulin Ca^2+^-binding proteins required for basal body genesis in cilia. The ciliary density of OSNs during the early postnatal stages were normal in *CETN2* mutant mice. However, massive cilia loss and disrupted structures were observed in mutant animals at P14 (and older). Impaired ciliary trafficking of olfactory signaling proteins, including Adcy3, probably caused these effects (Ying et al., 2014). Coincidentally, genetic ablation of the *GOOFY* gene (a Golgi protein specifically expressed in OSNs) in mice resulted in shortened olfactory cilia because of impaired intracellular trafficking of Adcy3 from the Golgi apparatus to olfactory cilia (Kaneko-Goto et al., 2013). Studies performed on *Caenorhabditis elegans* similarly demonstrated that the cilia structures of AWB neurons could be modified by the activation of sensory signaling cascades (Mukhopadhyay et al., 2008). Taken together, these reports strongly support our finding that Adcy3 expression in olfactory cilia is required for olfactory ultrastructure maintenance in postnatal OSNs. The information increases the possibility that a feedback interaction exists between olfactory ciliary structure maintenance and proteins involved in olfactory signaling (McEwen et al., 2008). However, our present study did not resolve whether disrupted ciliary structure is sufficient to induce neuronal loss. Alternatively, disruption of ciliary structure may be a consequence of OSN degeneration. Therefore, this work is an interesting starting point for future investigation.

Taken together, our study firmly demonstrated that Adcy3 is indispensable for OSN maturation and maintenance of olfactory cilium ultrastructure from postnatal stages until adulthood in mice.

## Author Contributions

ZZ, DY, and ZW conceived and designed the research; ZZ, DY, and MZ performed the experiments; DS and YZ contributed the reagents, materials, and analytic tools; ZZ, DY, NZ, and YZ analyzed the data; ZZ, DY, and ZW wrote the paper.

## Conflict of Interest Statement

The authors declare that the research was conducted in the absence of any commercial or financial relationships that could be construed as a potential conflict of interest.
